# Potential mouse models of coronavirus-related immune injury

**DOI:** 10.3389/fimmu.2022.943783

**Published:** 2022-09-02

**Authors:** Fu-Yao Nan, Cai-Jun Wu, Jia-Hui Su, Lin-Qin Ma

**Affiliations:** ^1^ Dongzhimen Hospital, Beijing University of Chinese Medicine, Beijing, China; ^2^ Department of Emergency Medicine, Dongzhimen Hospital, Beijing University of Chinese Medicine, Beijing, China; ^3^ Institute of Sepsis, Beijing University of Chinese Medicine, Beijing, China

**Keywords:** COVID-19, coronavirus, immune injury, mouse model, SARS-CoV-2

## Abstract

Basic research for prevention and treatment of coronavirus disease 2019 (COVID-19), caused by severe acute respiratory syndrome coronavirus 2 (SARS-CoV-2), continues worldwide. In particular, multiple newly reported cases of autoimmune-related diseases after COVID-19 require further research on coronavirus-related immune injury. However, owing to the strong infectivity of SARS-CoV-2 and the high mortality rate, it is difficult to perform relevant research in humans. Here, we reviewed animal models, specifically mice with coronavirus-related immune disorders and immune damage, considering aspects of coronavirus replacement, viral modification, spike protein, and gene fragments. The evaluation of mouse models of coronavirus-related immune injury may help establish a standardised animal model that could be employed in various areas of research, such as disease occurrence and development processes, vaccine effectiveness assessment, and treatments for coronavirus-related immune disorders. COVID-19 is a complex disease and animal models cannot comprehensively summarise the disease process. The application of genetic technology may change this status.

## Introduction

Coronaviruses are large, enveloped, positive-sense, single-stranded RNA viruses. They can infect a variety of vertebrates, including mammals, such as humans, and poultry, and cause multisystem diseases of the respiratory tract, liver, and gastrointestinal tract. Since late 2019, the coronavirus disease 2019 (COVID-19) pandemic has affected 216 countries and endangered the health of more than 200 million people, making it one of the most serious infectious diseases (1). Alpha, Beta, Gamma, Delta, Omicron, and other severe acute respiratory syndrome coronavirus 2 (SARS-CoV-2) variants (2) have emerged and are significantly impacting human society, economy, and life. Increasing attention is now focused on the prevention and treatment of viral pneumonia caused by SARS-CoV-2. The host immune-response disorder caused by SARS-CoV-2 is a key factor in the damage to multiple systems, including the lungs (3); however, the effects of prevention and treatment methods are difficult to evaluate. In this regard, it is also difficult to conduct extensive drug trials as the virus is highly infectious. Therefore, an effective animal model is useful for coronavirus-related immune injury research. In particular, an increasing number of autoimmune diseases related to COVID-19 infection have been reported recently, which has drawn more attention to the immune system damage caused by the imbalance of inflammatory factors after SARS-CoV-2 infection (4, 5). Previous reports of multiple cases of immune system damage after COVID-19 have revealed that very little is known about the complexity of COVID-19, and a reliable animal model of coronavirus-associated immune damage is necessary to support related research. Mice are widely used in biomedical research owing to their small size, ease of use, rapid reproduction, inbreeding ability, and ease of genetic modification. Here, we reviewed studies using mouse models of coronavirus pneumonia and explored potential mouse models of immune injury caused by coronavirus pneumonia. The findings may not provide a basis for experimental research on drugs for the prevention and treatment of COVID-19, but they do provide a foundation for the research on the physiological mechanisms and pathological damages of COVID-19.

## Mouse hepatitis virus strain-A59 mouse model

Mouse hepatitis virus strain-A59 (MHV-A59) and various coronaviruses have similar structures, which overlap well in multiple domains and show significant overall structural homology (6). The infectivity of coronaviruses may be closely related to fusion peptides (7), which participate in binding cell receptors and induce fusion between the virus and cell membrane. This structure also exists in MHV-A59, which is beneficial for the establishment of a mouse virus infection-related model. Therefore, fusion peptides in MHV-A59 are research candidates for antiviral drugs and candidate vaccines (8).

In addition, MHV-A59 and coronaviruses follow a similar pathological course after infection. Recently, evidence has shown that MHV-A59 infection can produce multisystem pathological processes, including autoimmune hepatitis-like disease, thymic degeneration, hypergammaglobulinemia, and temporary nerve demyelination (9). The respiratory tract and lung tissue may also be infected with MHV-A59, resulting in severe pathological damage, similar to the acute inflammation caused by SARS-CoV (10).

MHV-A59 is pneumophilic when mice are inoculated nasally, and it reproduces several clinical features of SARS-CoV-2 infection, including increased systemic inflammation in the heart, adipose tissues, and hypothalamus, as well as neutrophilia (10, 11). SARS-CoV-2 infection replacement models were successfully established using both C57BL/6J and BALB/c mice. Within 24 h from the viral infection, the mice developed symptoms such as increased body temperature, decreased activity or restlessness, accelerated breathing, weight loss, hypoxemia, anorexia, quadriplegia, and abdominal muscle spasms. Therefore, MHV-A59 can be used as a surrogate for SARS-CoV-2. Currently, multiple researchers have selected MHV-A59 as an alternative model for SARS-CoV-2 and have achieved good results with a wide range of systemic effects. Respiratory distress syndrome also develops in addition to the induction of hepatitis and encephalomyelitis by MHV-A59. All other MHV strains require a background of A/J or type-I interferon (IFN) deficiency to lead to serious diseases (12). In addition, some researchers have used ageing mice and MHV-A59 to simulate severe symptoms after COVID-19 infection, including up to 30% weight loss, anorexia, decreased oxygen saturation, and a series of pathological changes in the lungs, including neutropenia, monocytosis, γδ T cell loss, lymphocyte decline, and increased circulating pro-inflammatory cytokines (11). These studies provide an impetus for further studies on virus replacement models of coronavirus pneumonia.

## Mouse-adapted SARS-CoV-2-related immune injury model

Variation in the amino acid sequence in the viral receptor-binding domain of mouse angiotensin-converting enzyme 2 (ACE2) renders mice unable to be SARS-CoV-2 hosts (13). Therefore, by modifying the spike protein of SARS-CoV-2, a mouse-adapted SARS-CoV-2 strain that binds to the mouse ACE2 can be obtained. For example, MASCp6 can induce inflammatory responses and moderate pneumonia in young and old mice (14), which can cause histopathological changes such as tracheal degeneration and alveolar inflammation; viral antigens can be detected in the trachea, bronchioles, and type II pneumocytes. Based on reverse genetics, researchers (15) have constructed a recombinant virus SARS-CoV-2 MA that can utilise mouse ACE2; this replicates in the upper and lower respiratory tracts of BALB/c mice of all ages, resulting in mild to moderate pneumonia.

Notably, these constructed SARS-CoV-2 strains, which are adapted to mouse ACE2, can better simulate the clinical and pathological manifestations of human infection with SARS-CoV-2; therefore, they can be used to study the efficacy of antiviral drugs. The mouse-adapted strains are less pathogenic and infectious to humans and more suitable for drug and related research with broader prospects.

## Inactivated or recombinant virus-related immune injury model

Inactivated whole-virus vaccines have the advantage of relatively easy mass production and stable expression of a conformation-dependent epitope (16, 17). However, the disadvantages of inactivated preparations include the risk that vaccine preparations contain infectious viruses, and the immune responses can lead to abnormal presentations (18). Some researchers found that an inactivated SARS-CoV vaccine was able to induce neutralising antibodies in healthy young mice. However, the inactivated vaccine failed to induce enough antibodies in aged mouse models to show clinical manifestations similar to human infection with SARS-CoV (including increased levels of SARS-CoV replication and pathological changes in lung tissues). After inoculation of aged BALB/c mice with recombinant SARS-CoV containing the mutant spike glycoprotein, severe lung injury similar to that of human diseases is reproduced, including diffuse alveolar injury, hyaline membrane formation, and death. Because these recombinant BALB/c mice show high-titre replication of the virus *in vivo*, liver damage, multifocal interstitial lymphocyte infiltration, and other complications, they can be used as a model to evaluate vaccine effectiveness or viral pneumonia-related effects (19). Another study showed that although a double-inactivated SARS-CoV vaccine can provide protection against fatal diseases in young mice after homologous and heterologous attacks, it underperforms in aged animal models, exhibits increased eosinophilic immunopathology in the lungs, and does not significantly prevent viral replication (20). In most of these studies, the evaluation index was the viral load in the mouse lung that reached a certain standard.

Vaccine-associated enhanced respiratory disease (VAERD) was observed in a subset of preclinical models of the SARS-CoV-2 vaccine. VAERD is a modified or more severe presentation of the disease involving the lower respiratory tract and is caused by a pathogenic infection following vaccination with the same pathogen. VAERD may cause post-infectious, possibly immune-mediated, systemic diseases (21). This may be related to the fact that the vaccine again induces the required pathway for viral infection but produces many low-quality and low-activity antibodies (22).

## Spike protein-associated immune injury model

SARS-CoV-2 triggers infection by attaching surface-exposed spike glycoproteins to the host cell receptors. Spike proteins are promising targets for inducing an immune response and providing protection. Therefore, continuous efforts to develop SARS-CoV-2 vaccines and treatments focus on spike proteins (23). Research on SARS-CoV-2 vaccines found that subunit vaccines based on the spike protein induced an immune response (24). In a study from 2020 (25), immunogens based on spike protein-associated multi-epitope proteins were inoculated into different mouse strains, and all multi-epitope proteins were found to be highly immunogenic and capable of inducing antigen-specific antibody responses. Responses included a specific CD8+ T cell response that may upregulate IFN-γ, which is associated with the inhibition of viral replication and enhanced antigen delivery. This may benefit further development of SARS-CoV-2 vaccines or therapies (26). In many related studies, spike protein-related polypeptide preparations were injected intraperitoneally into C57BL/6J and BALB/c mice, and serum antibody concentrations and multiple cytokines were observed at different times (23, 27). This research on spike protein-related models has provided a basis for the development of vaccines and treatments for SARS-CoV-2 infections.

## mRNA vaccine-related immune injury model

Studies on coronaviruses have shown that mRNA vaccines can effectively elicit both humoral and cellular immunities. For SARS-CoV-2, the mRNA vaccines widely used worldwide include BNT162b2 (Pfizer- BioNTech) and mRNA-1273 (Moderna, Inc.). mRNA can effectively convey antigen expression and has good antigenicity. Researchers injected mRNA-1273 intramuscularly into multiple strains of mice and found that mRNA-1273 is a potent immunogen; a single dose can stimulate immunity and induce pseudo virus-neutralising antibody responses (28). Furthermore, intramuscular injection of low-dose mRNA-1273 in mice can effectively increase CD4+ T lymphocytes and germinal centre B cells, activate related immune pathways, and simultaneously activate IFN-γ and interleukin (IL)-4. This results in an appropriate ratio of IgG2a/IgG1 with good neutralising activity.

Abnormal immune responses caused by mRNA vaccines mostly manifest as allergic reactions to the active ingredients of the vaccine itself or to other components of the vaccine related to the lipid-based nanoparticle carrier. Therefore, mRNA vaccines are relatively safe and effective in inducing antibodies, but they can induce immune disorders related to antiviral protection and even clinical manifestations of VAERD in rare cases (22, 29). This might be because the mRNA vector has no characteristics of antibiotic resistance, genomic integration, or a strong immunogenic response (29). However, the mRNA vaccine BNT162b2 has an increased risk of causing multisystem inflammatory syndrome in minors (30), with symptoms including high fever, hypotension, weakness, pericardial effusion, elevated C-reactive protein/brain natriuretic peptide/troponin T/D-dimer ratio, and cardiac involvement. To the best of our knowledge, the abnormal respiratory symptoms caused by the mRNA vaccine have not been investigated, and research on the underlying immune mechanism is still insufficient.

## Lipopolysaccharide (LPS)-induced immune injury model

Administration of LPS to the airways causes inflammatory damage to the lungs (31). LPS is a component of the cell wall of gram-negative bacteria and is one of their main pathogenic factors. LPS is composed of a O-antigen, core polysaccharide, and lipid A, which is the main centre of toxicity and the main bioactive part of LPS. Therefore, LPS is widely used in animal models of lung injury (32, 33). C57BL/6J and BALB/c mice are often chosen as animal models. However, macrophages mainly exist in the liver and spleen, which means that LPS in the blood is mostly deposited in these organs after systemic administration, and the pulmonary inflammatory response and resultant damage are not as obvious in mice as in humans. Therefore, oral, nasal, or tracheal administration and inhalation are preferable administration methods in mice because they are more likely to cause inflammatory damage to the lungs and accumulation of cytokines compared to that seen with systemic administration (31). The American Thoracic Society recommends that acute inflammatory injury in the lung should be observed within 24 h after stimulation to distinguish it from chronic and sub-chronic lung injury (34). Most studies also performed acute lung injury studies within 24 h from LPS stimulation, as lung inflammation and immune responses are significantly elevated during this period (35, 36).

LPS-induced and virus-related lung injury may activate a common pathway, namely the Toll-like receptor (TLR)-related pathway. Therefore, the LPS-induced mouse lung injury model holds significance for the study of virus-related lung injury (37). Normal activation of TLRs is important for the human body to prevent microbial infections, including bacterial and viral. Excessive activation can lead to chronic inflammatory diseases such as diabetes and autoimmune diseases, whereas insufficient activation can lead to infectious diseases. The inflammatory response and autoimmune damage caused by LPS may be related to its effect on the TLR-dependent MyD88/NF-κB signalling pathway (38, 39).

## Conclusions and prospects

We reviewed mouse models of coronavirus-related immune injury, which are summarised in **Table 1**. Through the horizontal analysis of various mouse models, we found different advantages and limitations among the coronavirus-related immune injury mouse models. First, the biosecurity risk. Live viruses, such as mouse hepatitis virus strain-A59 or mouse-adapted SARS-CoV-2, present a structure similar to SARS-COV-2 and induce immunological and pathological damages comparable to SARS-COV-2 infection. However, these models are accompanied by a low but non-zero probability of infection in humans and other mammals, requiring higher laboratory biosafety levels. Second, genetic mutations may affect the stability of the models. The continuous mutation of key residues may have played a pivotal role in the ACE2 receptor modification in the newly prevalent SARS-COV-2 mutant (40). Gene mutations may change the structure of key proteins, such as the spike protein and ACE2, and affect immune responses, influencing the evaluation and establishment of mouse models with a mouse-adapted SARS-CoV-2 and spike protein. Similarly, the mRNA COVID-19 vaccine can effectively and stably cause immune damage in mice, but the risk of gene mutation is unpredictable (41). Third, inactivated or recombinant coronaviruses as well as LPS are easily obtained but present some disadvantages. Inactivated or recombinant coronaviruses cause different degrees of immune damage in mice of different ages, which may affect the stability of the mouse model (19, 20). LPS can induce severe immune damage similar to that of coronaviruses in the mouse lung, but since the structure of LPS and the coronavirus differs, it is difficult to simulate the exact pathological process (29, 30, 42).

**Table 1:** Comparison of different mouse models of coronavirus-related immune injury.

	Researchers	Mouse	Influence Factor	Indicators
**Mouse hepatitis virus strain-A59 mouse model**	Cowley TJ, Long SY, Weiss SR et al. (9)	4- to 5-week-old male C57BL/6 mice	A59/JHM recombinant viruses	IFN-γ, viral titres, CD8+ T cells and CD4+ T cells in liver and brain cells
	Zhao Z, Xiao Y, Xu L et al. (10)	6-week-old female BALB/c mice	MHV-A59 virus	IL-6, IFN-γ, TNF-α, IP-10, TGF-β, and MCP-1
	Ryu S, Shchukina I, Youm YH et al. (11)	2–6 months old and 20–24 months old male C57BL/6 mice	MHV-A59 virus	CD4+ T cell, CD4/CD8 T cell ratio, γδ T cell, and neutrophils in the lungs
**Mouse-adapted SARS-CoV-2-related immune injury model**	Sanclemente-Alaman I, Moreno-Jiménez L, Benito-Martín MS et al. (14)	9-month-old and 6-week-old BALB/c mice	SARS-CoV-2 MASCp6 virus	TNF-α, IL-1β, IL-6, IL-5, MCP-1, G-CSF, and pulmonary tissue viral load
	Dinnon KH, Leist SR, Schäfer A et al. (15)	12-month-old BALB/c mice	SARS-CoV-2 MA virus	Body weight, lung function, and bronchiolar or alveolar pathology
**Inactivated or recombinant virus-related immune injury model**	Rockx B, Sheahan T, Donaldson E et al. (19)	6-week-old female BALB/c mice and 12-month-old female BALB/c mice	Recombinant SARS-CoV Urbani strain	Viral titres in tissue samples, bronchiolar or alveolar pathology
	Bolles M, Deming D, Long K et al. (20)	6 to 8 weeks old female BALB/c mice and 12 to 14 months old female BALB/c mice	Double-inactivated SARS-CoV vaccine	Viral titres of lungs, tissue damage and characterization of inflammation in the lungs, eosinophils, IL-4,IL-5, IFN-γ, antigen-specific IgG
**Spike protein-associated immune injury model**	Vishwakarma P, Yadav N, Rizvi ZA et al. (23)	7–8-week-old female BALB/c mice	SARS-CoV-2 spike glycoprotein	Peptide-specific IgG, CD8+ T cells
	Shrivastava T, Singh B, Rizvi ZA et al. (27)	7–8-week-old male C57BL/6 mice	SARS-CoV-2 spike protein	IgG subclass, CD4+ T cells, CD8+ T cells, IFN-γ, IL-17
**mRNA vaccine–related immune injury model**	Corbett KS, Edwards DK, Leist SR et al. (28)	6-week-old female BALB/cJ, C57BL/6J, and B6C3F1/J mice	mRNA-1273, an mRNA vaccine of SARS-CoV-2	Peptide-specific IgG, IFN-γ, IL-4, IL-5, IL-13, CD4+ T cells, CD8+ T cells
**Lipopolysaccharide (LPS)-induced immune injury model**	Zhou Y, Li P, Goodwin AJ et al. (31)	7–8-week-old CD-1 outbred mice	Intratracheal instillation of LPS	BALF cell number, TNF-α, IL-6, IL-1β, IFN-γ, MIP-1, lung tissue myeloperoxidase activity
	Zhou M, Fang H, Du M et al. (32)	8–10-week-old myeloid-specific PTEN knock-in mice	Intratracheal instillation of LPS	TGF-β, TNF-α, IL-1β, IL-17A, IL-23, myeloperoxidase activity
	Toki S, Zhou W, Goleniewska K et al. (33)	9–13-week-old female C57BL/6 mice	Intranasally challenged with LPS	6-keto-PGF_1α_, TNF-α, IL-1α, IL-1β, IL-6, IL-10, mouse myeloperoxidase

IFN, interferon; IL, interleukin; TNF-α, tumour necrosis factor α; IP-10, inducible protein 10; TGF-β, transforming growth factor β; MCP-1, monocyte chemotactic protein 1; IgG, immunoglobin G; BALF, bronchoalveolar lavage fluid; MIP-1, macrophage inflammatory protein 1; 6-keto-PGF_1α_, 6 keto prostaglandin F_1α_.

As COVID-19 continues to spread worldwide, its scope of influence is increasing, and there are increasing scientific problems related to viral pneumonia. It is even more necessary to use scientific animal models as the basis for research in viral pneumonia and related lung injury. The establishment of correct models can improve the speed of clinical trials and ensure their effectiveness. We have chosen the most widely used mouse models for discussion, but there are other animal models besides mice. Recently, researchers successfully used multiple SARS-CoV-2 variants, including Omicron, to cause pathological damage to the lung of a hamster, in which the virus replicated at high titres (43). Ferrets are widely used in respiratory studies, and inoculation with SARS-CoV-2 through the nose can cause pathological damage to the lungs (44). Other models such as cats, minks, and rhesus monkeys can be successfully infected with SARS-CoV-2, causing the related pathological damage (45, 46). However, no single animal model can reproduce the overall pathogenesis or predict autoimmune responses as accurately as humans. Animal models also cannot fully reproduce the multisystem damage caused by virus invasion in humans due to multisystem interactions. In addition, animal models differ from humans in their physiological structure, molecular biological pathways, and their susceptibility to viruses, warranting further research.

Transgenic technology may change this situation (47). Recently, researchers proposed CAG promoter-driven human ACE2-transgenic mouse models, which, to some extent, reproduce the immune response and pathological damage after COVID-19 infection (48). Additionally, a mouse model expressing human ACE2, which was generated using CRISPR/Cas9 knock-in technology, was infected with SARS-CoV-2, developed interstitial pneumonia, and had elevated cytokines (49). In the future, the application of transgenic technology may provide accessible immune injury models, which will not only be applied to research studies on autoimmune-related diseases but also contribute to the establishment of an accessible and standardised animal model library (50). Different characteristics of these animal models could be explored by researchers in various pathologic injuries, in turn enhancing study reproducibility.

## Author contributions

F-YN: Original draft preparation and literature research. C-JW: Conceptualization and fund supporting. J-HS: Proofreading and revising. L-QM: Reviewing and editing. All authors contributed to the article and approved the submitted version.

## Funding

The study was funded by the Capital Medical Development Scientific Research Fund (2020-2-4192) and the Science and Technology Innovation Project of Dongzhimen Hospital (DZMKJCX-2020-027).

## Conflict of interest

The authors declare that the research was conducted in the absence of any commercial or financial relationships that could be construed as a potential conflict of interest.

## Publisher’s note

All claims expressed in this article are solely those of the authors and do not necessarily represent those of their affiliated organizations, or those of the publisher, the editors and the reviewers. Any product that may be evaluated in this article, or claim that may be made by its manufacturer, is not guaranteed or endorsed by the publisher.

## Abbreviations

ACE2, angiotensin-converting enzyme 2; COVID-19, coronavirus disease 2019; IFN, interferon; LPS, lipopolysaccharide; MHV-A59, mouse hepatitis virus strain-A59; SARS-CoV, severe acute respiratory syndrome coronavirus; SARS-CoV-2, severe acute respiratory syndrome coronavirus 2; TLR, Toll-like receptor; VAERD, vaccine-associated enhanced respiratory disease.
